# Supplementary material for: An experimentally validated manually curated genome-scale metabolic model of Enterococcus faecium TX0016 reproduces high-throughput phenotyping data
Source: Front Microbiol. 2026 Oct 9;17:1941652. doi: 10.3389/fmicb.2026.1941652 (PMC13691439; doi:10.3389/fmicb.2026.1941652)
Supplement: Supplementary file 1 [file Data_Sheet_1.PDF]

An experimentally validated manually curated genome-scale metabolic model of *Enterococcus faecium* TX0016 reproduces high-throughput phenotyping data

Dina Samir Rasmi, Jayanth Krishnan, Yomna A. Hashem, Bernhard O. Palsson, Mona T. Kashef, Jonathan M. Monk, Ramy K. Aziz

SUPPLEMENTARY MATERIAL

- Supplementary Figures S1 and S2
- Supplementary Text
- Supplementary Tables S10 and S11

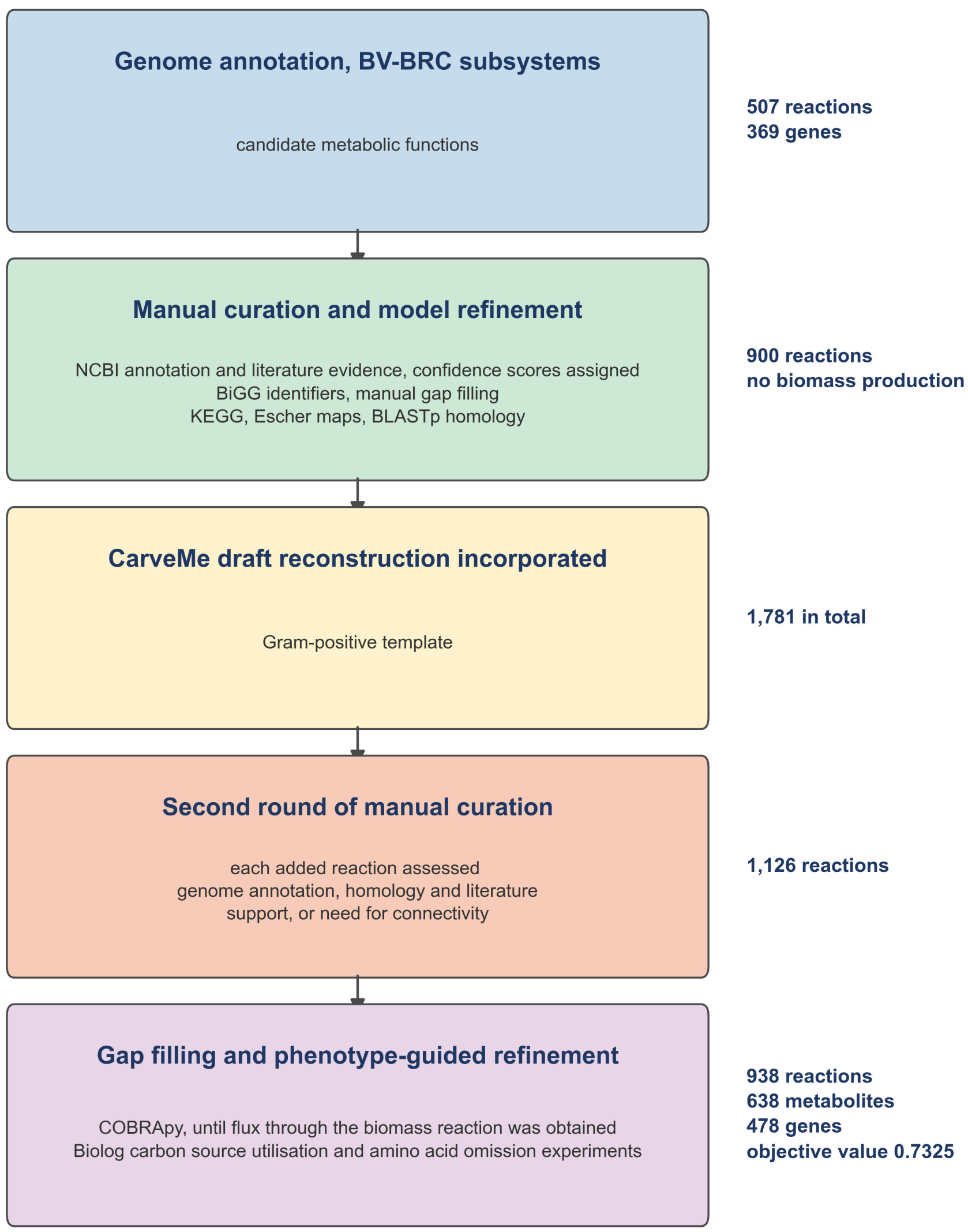

Figure S1: Major stages of iDR478 reconstruction

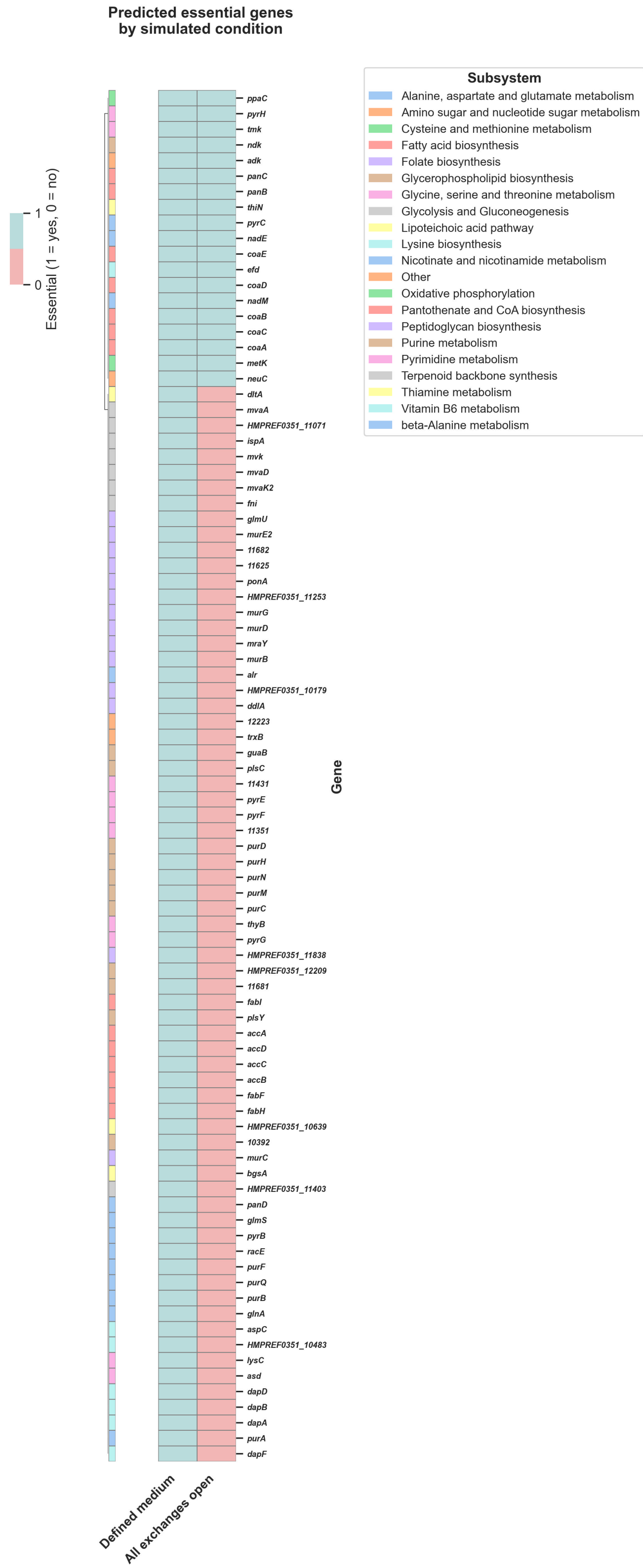

Figure S2: Color-coded matrix of the predicted essential genes in defined medium and whenexchanges are open with their corresponding subsystem

Supplementary Text

Quantitative history of the reconstruction workflow (Figure S1)

The initial set of reactions comprised 507 candidate metabolic functions drawn from 369 genes from the BV-BRC subsystems. Manual curation against the NCBI and KEGG annotations, gap-filling of pathways using the Escher maps, assigning BiGG identifiers brought the curated draft to 900 reactions, at which point the model could not produce biomass. To resolve network gaps and ensure model feasibility, we curated the draft GEM using the gap filling algorithm implemented in COBRApy v 0.25.0, and the BiGG Models database, downloaded on August 1, 2023, as the universal database of candidate biochemical reactions. The COBRApy *gapfilling* module uses mixed-integer linear programming to compare the draft model against the universal database. We performed gapfilling by constraining the model to a defined media input and setting biomass production as the biological objective function. Based on these constraints, the algorithm identified the minimal set of external reactions required from the universal database to achieve simulated growth. The proposed reaction sets were subsequently reviewed and integrated into the network to produce a functionally complete model.Reactions from a CarveMe draft reconstruction of the same genome, generated with the Gram-positive template, were also incorporated, giving 1,781 in total. Each added reaction was evaluated with the same approach of intensive manual curation, and thus reactions with no genome annotation, no homology, or no literature support were removed. Only transporters needed for network connectivity were retained, reducing the reconstruction to 1,000 reactions. The model was again curated to remove redundancy and useless reactions, and finally validated with experimental results to attain 938 reactions.

Comparing the CarveMe reaction set with the final deposited model highlights 504 reactions that have been contributed by the CarveMe set. Of these, 278 carry a GPR association; 244 are supported by genome annotation; and 31 by homology search, while 226 have no annotation recorded and predominantly are transport and exchange reactions.

Comparison between iDR478 and available semi-automated *E. faecium* reconstructions (Additional text to section 3.6)

Subsystem counts are not directly comparable between the reconstructions owing to identifier mismatches and reflect differences in labelling convention rather than coverage. Reactions in iDR478 were assigned to KEGG pathways during curation and are reported under those names, whereas the AGORA2 reconstructions carry a different set of labels. For example, they separate purine and pyrimidine synthesis from catabolism, carries four distinct labels for unclassified reactions, and includes categories not expected in enterococci, among them cholesterol and bile acid metabolism, lipopolysaccharide biosynthesis and heparan sulfate degradation. These are likely results of the automated construction process.

None of the three biomass formulations was determined experimentally for *E. faecium*, and all three derive from formulations published for other organisms. The iDR478 biomass reaction was assembled from two formulations for Gram-positive organisms: *E. faecalis*, the closely related enterococcus, and *Bacillus*, the model Gram-positive organism of CarveMe. The biomass formulation comprises 45 components: all 20 amino acids, ATP, GTP, the ribonucleotides and deoxyribonucleotides, six cofactors (biotin, CoA, NAD, pyridoxal 5-phosphate, THF and thiamine diphosphate), enterococcal cell envelope from *E. faecalis*: peptidoglycan, wall teichoic acid, lipoteichoic acid, cardiolipin and undecaprenyl pyrophosphate, together with a separate ATP maintenance reaction constrained at 0.81 mmol $gDW^{-1}h^{-1}$.The AGORA2 biomass reactions derive from the KBase draft reconstructions and were subsequently adjusted for class-specific membrane and cell wall structures.. That for strain DO comprises 101 components and that for TX1330 comprises 64, and the two differ from one another in the representation of phospholipids, porphyrins, and polyamines, components.. Both include DNA, RNA, and protein synthesis as lumped polymerization reactions, eleven metals, and a broad cofactor set. The DO reconstruction additionally includes ubiquinone-8, which enterococci do not synthesise, using menaquinone instead. Comparison across the AGORA2 resource shows that the same 100-component formulation recurs in reconstructions of *Enterococcus, Lactococcus,* and *Streptococcus* species, so the difference between the two *E. faecium* reconstructions probably reflects the template each received rather than strain-level biology. Experimental determination of the biomass composition of *E. faecium* would improve all three reconstructions and is among the most useful measurements that could be made for constraint-based modelling of this organism.

Supplementary Table S10: Comparison of model characteristics of the iDR478 and AGORA2 reconstructions

Criterion	iDR478 DO	AGORA2 DO	AGORA2 TX1330
**Number of genes**	478	620	783
**Total number of reactions**	938	1014	1074
**Biomass**	1	1	1
**Metabolism**	582	715	748
**Transport**	201	149	167
**Exchange**	154	149	158
**Blocked reactions**	99 (10.6%)	304 (30%)	408 (38 %)
**Number of Metabolites**	638	917	936
**Subsystem labels**	62	73	75
**GPR metabolic reactions**	98.8% (575/582)	81.5% (583/715)	94.5% (707/748)
**GPR, transporters**	41/201	95 of 149	122 of 167
**Reaction confidence scores**	1 to 3, per reaction	_	_
**Curation**	Manual, reaction level	Semi-automated (DEMETER*)	Semi-automated (DEMETER*)

Table S11: Predicted amino acid auxotrophies under the complex medium distributed with the DEMETER pipeline, growth threshold 0.01 h^{-1}

Amino acid	Experiment (DO)	iDR478 (DO)	AGORA2 DO	AGORA2 TX1330
**L-Alanine**	prototroph	prototroph	auxotroph	prototroph
**L-Arginine**	auxotroph	auxotroph	auxotroph	auxotroph
**L-Asparagine**	prototroph	prototroph	auxotroph	no exchange
**L-Aspartate**	prototroph	prototroph	no exchange	no exchange
**L-Cysteine**	inconclusive	prototroph	no exchange	no exchange
**L-Glutamate**	prototroph	prototroph	prototroph	prototroph
**L-Glutamine**	prototroph	prototroph	auxotroph	auxotroph
**Glycine**	inconclusive	prototroph	prototroph	prototroph
**L-Histidine**	auxotroph	auxotroph	auxotroph	auxotroph
**L-Isoleucine**	auxotroph	auxotroph	auxotroph	auxotroph
**L-Leucine**	auxotroph	auxotroph	auxotroph	auxotroph
**L-Lysine**	prototroph (adapted)	prototroph	no exchange	auxotroph
**L-Methionine**	auxotroph	auxotroph	prototroph	prototroph
**L-Phenylalanine**	prototroph (adapted)	prototroph	auxotroph	prototroph
**L-Proline**	prototroph	prototroph	prototroph	prototroph
**L-Serine**	inconclusive	prototroph	auxotroph	prototroph
**L-Threonine**	auxotroph	auxotroph	auxotroph	auxotroph
**L-Tryptophan**	auxotroph	auxotroph	auxotroph	auxotroph
**L-Tyrosine**	prototroph (adapted)	prototroph	auxotroph	prototroph
**L-Valine**	auxotroph	auxotroph	auxotroph	auxotroph
**Auxotrophies predicted**	8	8	13	9
**Agreement with experiment**	—	20 of 20	9 of 17	12 of 17

Comparison of carbon source utilization

AGORA2 reports validation for DO against phenotypic datasets for both strains. For DO, 39 of 51 observations from NJC19 and BacDive were correctly predicted **(Supplementary Table S13)**and for TX1330, 14 of 20 observations, all from NJC19 **(Supplementary Table S14)**. Seventeen of the DO substrates and 8 of the TX1330 substrates were also tested in our Biolog panel. Among the DO substrates, 13 out of the 17 agree with our model's predictions and experiments. D-tagatose is recorded as utilized by NJC19 and as not utilized by BacDive. Discrepancies were also reported in D-sorbitol and D-melibiose and sucrose. The remaining 63 out of 80 Biolog substrates evaluated in our study have no entry for *E. faecium* in the AGORA2 validation data.

However, given that the two assessments use different endpoints, they are not directly comparable. AGORA2 evaluated whether the corresponding exchange reaction could carry flux under an unlimited-medium formulation, while, in iDR478, each substrate was supplied individually in a CDM lacking glucose, and growth was compared for each substrate against the yield of growth in the same CDM without glucose or any carbon source (growth threshold of 0.07).

The two datasets, NJC19 and BacDive, used for validating the carbon source utilization in AGORA2 reconstructions disagree with each other on D-tagatose, being recorded as utilized by NJC19 and as not utilized by BacDive for the DO strain, with the difference in phenotypic databases demonstrating the need for strain-specific experimental data. In our experimental Biolog screens, DO was able to utilize D-tagatose as carbon source, and this finding was used to refine the iDR478 model. The model includes the tagatose pathway through a tagatose-6-phosphate kinase (HMPREF0351_11049, confidence score 2) and a PTS transporter added at confidence score 1, but retained based on the phenotypic result.

NJC19 and BacDive also disagree with Biolog carbon utilization experiments on D-sorbitol, D-melibiose, and sucrose. D-sorbitol and D-melibiose are marked as not utilized in BacDive, but are utilized in the Biolog screens. Both iDR478 and the AGORA2 reconstructions predicted the utilization of D-sorbitol, agreeing with Biolog validation. D-melibiose utilization, however, was not predicted in the AGORA2 models but was utilized by the iDR478 model through the broad spectrum alpha-galactosidase, HMPREF0351_11597, assigned in KEGG annotation as "melibiase."

Sucrose shows the converse: BacDive records utilization, our assay did not, and iDR478 predicts growth through an annotated β-fructofuranosidase (HMPREF0351_11791) we previously discussed, whose corresponding enzyme is absent from the AGORA2 DO reconstruction, again. The discordance in sucrose utilization was previously described here in the model and again may be due to inducible expression or inter-species differences, highlighting again the importance of strain-specific experimental testing and manual refinement of the model.
